# BP-ANN for Fitting the Temperature-Germination Model and Its Application in Predicting Sowing Time and Region for Bermudagrass

**DOI:** 10.1371/journal.pone.0082413

**Published:** 2013-12-13

**Authors:** Erxu Pi, Nitin Mantri, Sai Ming Ngai, Hongfei Lu, Liqun Du

**Affiliations:** 1 College of Life and Environmental Science, Hangzhou Normal University, Hangzhou, Zhejiang, PR China; 2 College of Life Science, Zhejiang Sci-Tech University, Hangzhou, Zhejiang, PR China; 3 School of Applied Sciences, Health Innovations Research Institute, RMIT University, Melbourne, Victoria, Australia; 4 Department of Biology, The Chinese University of Hong Kong, Shatin, Hong Kong SAR; Lawrence Berkeley National Laboratory, United States of America

## Abstract

Temperature is one of the most significant environmental factors that affects germination of grass seeds. Reliable prediction of the optimal temperature for seed germination is crucial for determining the suitable regions and favorable sowing timing for turf grass cultivation. In this study, a back-propagation-artificial-neural-network-aided dual quintic equation (BP-ANN-QE) model was developed to improve the prediction of the optimal temperature for seed germination. This BP-ANN-QE model was used to determine optimal sowing times and suitable regions for three *Cynodon dactylon* cultivars (*C. dactylon*, ‘Savannah’ and ‘Princess VII’). Prediction of the optimal temperature for these seeds was based on comprehensive germination tests using 36 day/night (high/low) temperature regimes (both ranging from 5/5 to 40/40°C with 5°C increments). Seed germination data from these temperature regimes were used to construct temperature-germination correlation models for estimating germination percentage with confidence intervals. Our tests revealed that the optimal high/low temperature regimes required for all the three bermudagrass cultivars are 30/5, 30/10, 35/5, 35/10, 35/15, 35/20, 40/15 and 40/20°C; constant temperatures ranging from 5 to 40°C inhibited the germination of all three cultivars. While comparing different simulating methods, including DQEM, Bisquare ANN-QE, and BP-ANN-QE in establishing temperature based germination percentage rules, we found that the R^2^ values of germination prediction function could be significantly improved from about 0.6940–0.8177 (DQEM approach) to 0.9439–0.9813 (BP-ANN-QE). These results indicated that our BP-ANN-QE model has better performance than the rests of the compared models. Furthermore, data of the national temperature grids generated from monthly-average temperature for 25 years were fit into these functions and we were able to map the germination percentage of these *C. dactylon* cultivars in the national scale of China, and suggested the optimum sowing regions and times for them.

## Introduction

*Cynodon dactylon* (Linnaeus) Persoon (Family: Poaceae, bermudagrass in English) is a perennial, creeping grass. Although it is widely found in the tropical and warm temperate regions, bermudagrass is predominantly distributed between 45° North and 45° South latitudes [1], [2]. Currently, *C. dactylon* is globally used as a turf grass, fodder and medicinal plant, and it was also used for removal of heavy metals from contaminated soils [3]–[5]. This grass is adapted to extremely variable environments, such as fertile fields, arid land, saline land, wet irrigation canals and even contaminated wastelands with high levels of Pb, Cd, Zn and Cu [6]–[10]. In addition, the extracts from bermudagrass are well known for various medicinal properties including antimicrobial [11], [12], anti-inflammatory [13], [14], immunomodulatory [15], [16] and anti-diarrhea activities [17]. It has been therefore used to treat traumatic wounding, kidney calculi [18], hypoglycemia [19], depression [20] and cancer [21]–[23].

Seed germination percentage is a major criterion used for evaluating suitability of an environment for grass cultivation. Previous studies showed that extreme temperatures could lead to seed dormancy and significantly decreased germination percentage [24]–[26]. Constructing a precise mathematical model that correlates the germination percentage with temperature may avoid failure of plantation due to inappropriate sowing timing or mismatch between the grass species and climate zone. Hence it would be very helpful for decision-makers to select grass species and sowing time for lawn, erosion control and forage cultivation. Using thermal time approach, Bradford [27] constructed temperature/water potential based seed germination and dormancy models. This study showed that germination experiments under temperature regime with discrete (stepwise) changes could also be used to accurately predict thermal responses of seeds in field environment with continuously changing temperature. Hardegree and Van Vactor [28], [29] used the Piece-wise linear (PWL) regression equations to confirms that constant-temperature experimental results derived mathematical models could be used to predict the germination/growth responses to the combined effects of multiple environmental factors in the field. Although various experimental procedures have been established to generate data for the development and validation of reliable prediction models [30]–[35], the R^2^ values of temperature-germination percentage functions in some of the previous studies were relatively low (0.61–0.80) [34], [36] and more accurate predictions are desired.

Recently, the Geographic Information System (GIS) based spatial prediction approach was introduced for the creation of quantitative and accurate grass suitability maps [36]. The grid data of mean minimum and maximum temperatures [37] were used for calculating suitability values of regions *via* the temperature-germination percentage functions. Therefore, the suitability of grass for different regions could be appropriately visualized on the map. However, the visualization of time scale (season) of these maps remains to be streamlined for readability to facilitate their practical application.

The objectives of this research were to: (i) explain the correlation error between temperature and seed germination percentage caused by selection of functions (ii) provide a new program for optimizing the temperature-germination function, (iii) use temperature-based seed germination percentage function in combination with national temperature grids in China for predicting the suitability of three bermudagrass cultivars, *C. dactylon*, ‘Savannah’ and ‘Princess VII’.

## Results

### Germination response to Diurnal Fluctuations of Temperature

The germination response of the three bermudagrass cultivars was similar (Tables 1–3). All the three cultivars were capable of germinating in warm-period temperature (T_2_) from 25 to 35°C and cool-period temperature (T_1_) ranging from 5–40°C. The optimal temperature for seed germination is defined as that which is not lower than the maximum germination minus one-half of its confidence interval (P = 0.05). For example, the maximum germination percentage (the optimal temperature for seed germination) of *C. dactylon* was 87.3% at 15/35°C (Table 1); and the germination percentage at 5/30°C was only 80.7%, but it was still accepted as the optimal temperature for seed germination since the one-half of its confidence interval was 7.0% and 80.7%>(87.3%–7.0%). Fluctuating between 5–25°C cool-period temperature and 30–40°C warm-period temperature gave rise to the optimal temperature for seed germination. On the other hand, germination percentage was usually lower than 50% at constant temperature ranging from 20 to 40°C (except the “Savannah” at constant 25/25°C in Table 2), for example the germination percentages of “Princess VII” (Table 1), were 0, 36.0%, 5.3%, 12.7% and 20.0% at constant temperature regimes of 20/20, 25/25, 30/30, 35/35 and 40/40°C, respectively. Interestingly, germination percentage of *C. dactylon* seed at a constant 30°C only reached 1.3%.

**Table 1:** Quadratic response surface based on estimated percent germination with confidence interval at the 0.05 probability level for seeds of the *C. dactylon.*

Cool periodtemperature (°C)16 h	Warm period temperature (°C) 8 h							
	5	10	15	20	25	30	35	40
	% Germination after 15–20 days							
5	0.0±0.0	0.0±0.0	0.0±0.0	0.0±0.0	28.0±7.2	80.7±7.0*	76.7±9.5*	21.3±4.2
10		0.0±0.0	0.0±0.0	0.0±0.0	36.7±3.1	82.7±8.1*	84.7±5.0*	42.0±6.9
15			0.0±0.0	0.0±0.0	44.0±4.0	82.0±3.5*	[87.3±8.3]	79.3±3.1*
20				0.0±0.0	4.7±1.2	59.3±8.1	79.3±7.0*	81.3±13.3*
25					4.7±1.2	5.3±1.2	52.7±8.1	58.7±5.0
30						1.3±1.2	1.3±1.2	7.3±3.1
35							5.3±3.1	13.3±5.0
40								3.3±2.3

Note: 1. Data presented are mean ± a half of the confidence interval as determined from regression equations. The maximum calculated germination is enclosed by brackets.

2. Means not lower than the maximum germination minus one-half of its confidence interval.

**Table 2:** Quadratic response surface based on estimated percent germination with the confidence interval at the 0.05 probability level for seeds of the cultivar ‘Savannah’.

Cool periodtemperature (°C)16 h	Warm period temperature (°C) 8 h							
	5	10	15	20	25	30	35	40
	% Germination after 15–20 days							
5	0.0±0.0	0.0±0.0	0.0±0.0	24.7.0±4.2	77.3±6.1	86.0±5.3*	91.3±2.3*	82.7±13.3*
10		0.0±0.0	0.0±0.0	34.7.0±3.1	83.3±4.2	88.0±3.5*	88.7±2.3*	80.7±3.1
15			0.0±0.0	33.3.0±4.6	83.3±3.1	71.3±4.2	92.7±1.2*	87.3±6.4*
20				13.7.0±6.4	46.0±9.2	81.3±4.2	83.3±11.4*	[94.0±3.5]
25					61.3±4.2	42.0±13.1	73.3±4.6	91.3±4.6*
30						36.7±7.0	66.0±5.3	40.0±3.5
35							49.3±8.3	60.0±4.0
40								35.3±5.0
5	0.0±0.0	0.0±0.0	0.0±0.0	24.7.0±4.2	77.3±6.1	86.0±5.3*	91.3±2.3*	82.7±13.3*

Note: 1. Data presented are mean ± a half of the confidence interval as determined from regression equations. The maximum calculated germination is enclosed by brackets.

2. Means not lower than the maximum germination minus one-half of its confidence interval.

**Table 3:** Quadratic response surface based on estimated percent germination with the confidence interval at the 0.05 probability level for seeds of the cultivar ‘Princess VII’.

Cool period temperature (°C) 16 h	Warm period temperature (°C) 8 h							
	% Germination after 15–20 days							
	5	10	15	20	25	30	35	40
5	0.0±0.0	0.0±0.0	0.0±0.0	0.0±0.0	54.7±1.2	91.3±2.3*	96.0±3.5*	66.0±13.1
10		0.0±0.0	0.0±0.0	0.0±0.0	76.7±4.6	[96.7±2.3]	95.3±1.2*	49.3±6.4
15			0.0±0.0	0.0±0.0	83.3±3.1	94.0±2.0*	94.7±1.2*	94.0±3.5*
20				0.0±0.0	16.0±2.0	84.7±7.0	[96.7±11.4]	92.7±3.1*
25					36.0±2.0	18.7±8.3	86.7±6.4	90.0±5.3*
30						5.3±5.8	18.0±2.0	16.0±12.5
35							12.7±5.8	34.7±6.1
40								20.0±7.2

Note: 1. Data presented are mean ± a half of the confidence interval as determined from regression equations. The maximum calculated germination is enclosed by brackets.

2. Means not lower than the maximum germination minus one-half of its confidence interval.

The maximum germination and the mean of germination of the three bermudagrass cultivars are presented in Table 4. The germination percentage of ‘Princess III’ appeared to be the highest (96.7%), but it was not significantly different (P>0.05) from that of the other two cultivars (87.3% and 94.0% for *C. dactylon* and ‘Savannah’, respectively). Depending on the cultivar, 25%–27.8% of the temperature regimes supported the optimal temperature for seed germination. Only eight temperature regimes, 30/5, 30/10, 35/5, 35/10, 35/15, 35/20, 40/15 and 20/40°C, supported the optimal temperature for seed germination for all three tested cultivars (Tables 1–4).

**Table 4:** Comparison of the temperature–germination profiles for the five bluegrass cultivars.

Germinationparameter	Sources		
	*C. dactylon*	Savannah	Princess VII
Profile mean	31.3	52.7	45.0
Regimes with somegermination	72.2	83.3	72.2
Maximum germination	87.3	94.0	96.7
Mean of somegermination*	43.3	63.2	62.3
Mean of optima	81.6	88.5	94.1
Regimes with optimumgermination	25.0	27.8	27.8
Profile mean	31.3	52.7	45.0

Note: *The mean of some germination refers to the mean value of regimes with any germination more than zero [47], [52].

### Performance of Different Regression Models

**Figure 1:**
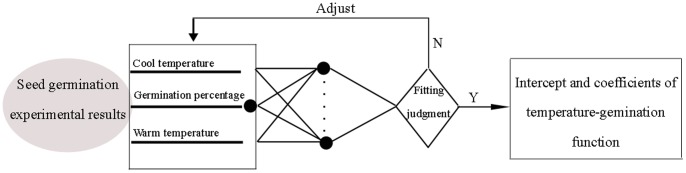
Schematic diagram of back propagation artificial neural networks used for fitting the regression functions of bermudagrass germination response to diurnal fluctuations of temperature.

**Figure 2:**
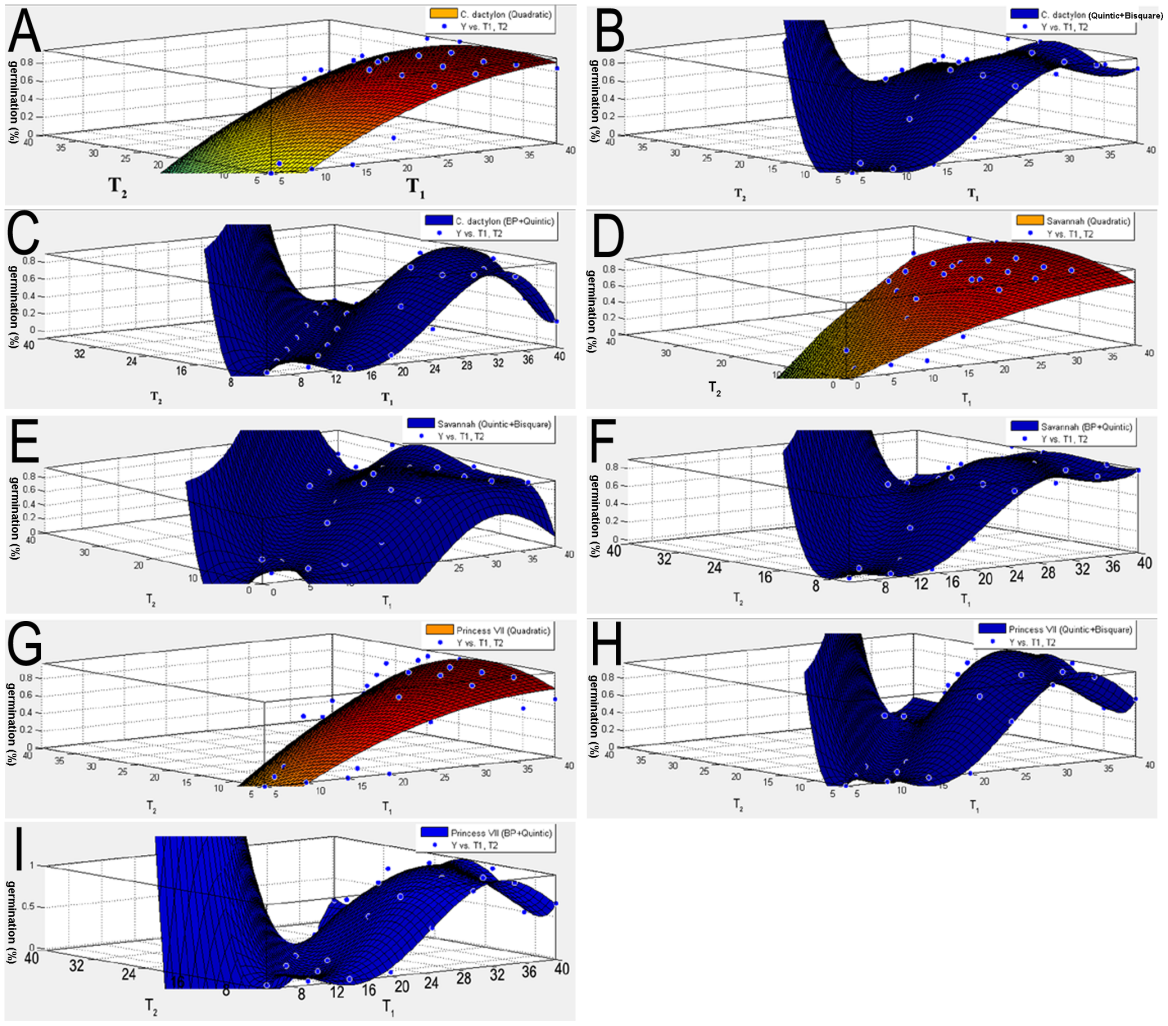
Three dimensional plot map of bermudagrass germination in which the estimated percent germination values in different temperature regimes were used for their constructions; (A–C) *C. dactylon*, (D–F) ‘Savannah’ and (G–I) ‘Princess VII’.

All the regression equations of temperature treatments that assess the germination suitability of the three bermudagrass cultivars are summarized in Table 5. Quadratic and quintic general equations were used for data simulation. The bisquare and BP-ANN approaches (Fig. 1) were utilized for optimizing these equation coefficients. There was no significant difference between the quintic functions and bisquare optimized quintic functions. Bisquare quintic functions of cultivars *C. dactylon*, ‘Savannah’ and ‘Princess VII’ showed lower R^2^ than quintic functions (data not shown). Similar optimization results were observed for all the cultivars tested. Generally, quintic equations performed better than quadratic equations (Fig. 2, Table 5). The highest R^2^ values were generated by the back propagation artificial neural networks aided dual quintic equation (BP-ANN-QE) model (0.9439 to 0.9813). In contrast, the dual quadratic equation model (DQEM) generated the lowest R^2^ values (0.6940 to 0.8177).

**Table 5:** Equations and R^2^ values for predicting germination as a function of cool (T_1_) and warm (T_2_) temperature treatments for the three *C. dactylon* cultivars tested.

Cultivars		R^2^	Equation
*C. dactylon*	Quadratic	0.8177	Y = −0.5856+0.06925*T_1_−0.006143*T_2_−0.0008586*T_1_^2^+0.0006574*T_1_*T_2_−0.0007847*T_2_^2^
	Quintic+Bisquare	0.9166	Y = 0.4026+0.02444*T_1_−0.133*T_2_−0.0291*T_1_^2^+0.05145*T^1^*T_2_−0.01535*T_2_^2^+0.002361*T_1_^3^−0.002139*T_1_^2^*T_2_−0.002076*T_1_*T_2_^2^+0.001802*T_2_^3^−6.199e-005*T_1_^4^−7.613e-006*T_1_^3^*T_2_+0.0002068*T_1_^2^*T_2_^2^−0.0001705*T_1_*T_2_^3^+2.974e-005*T_2_^4^+5.357e-007*T_1_^5^+5.886e-007*T_1_^4^*T_2_−2.712e-006*T_1_^3^*T_2_^2^+<1?show=[sr]?>1.219e-006*T_1_^2^*T_2_^3^+1.018e-006*T_1_*T_2_^4^−5.966e-007*T_2_^5^
	Quintic+BP-ANN	0.9813	Y = −0.2789+15.74*T_1_−11.43*T_2_−88.16*T_1_^2^+31.27*T^1^*T_2_+34.83*T_2_^2^+180.4*T_1_^3^+0.5264*T_1_^2^*T_2_−128.8*T_1_*T_2_^2^−3.322*T_2_^3^−139*T_1_^4^−163.4*T_1_^3^*T_2_+510.2*T_1_^2^*T_2_^2^−462.2*T_1_*T_2_^3^+206.5*T_2_^4^+32.55*T_1_^5^+124.5*T_1_^4^*T_2_−312.5*T_1_^3^*T_2_^2^+248.7*T_1_^2^*T_2_^3^−16.93*T_1_*T_2_^4^−59.17*T_2_^5^
Savannah	Quadratic	0.7617	Y = −0.5168+0.06924*T_1_−0.01989*T_2_−0.000934*T_1_^2^+0.001138*T_1_*T_2_−0.0007981*T_2_^2^
	Quintic+Bisquare	0.9301	Y = 0.3523+0.04868*T_1_−0.1349*T_2_−0.0311*T_1_^2^+0.04712*T^1^*T_2_−0.01236*T_2_^2^+0.002363*T_1_^3^−0.001472*T_1_^2^*T_2_−0.002595*T_1_*T_2_^2^+0.001872*T_2_^3^−6.187e-005*T_1_^4^−9.422e-006*T_1_^3^*T_2_+0.0001502*T_1_^2^*T_2_^2^−<1?show=[sr]?>7.953e-005*T_1_*T_2_^3^−9.526e-006*T_2_^4^+5.348e-007*T_1_^5^+5.833e-007*T_1_^4^*T_2_−2.575e-006*T_1_^3^*T_2_^2^+2.423e-006*T_1_^2^*T_2_^3^−1.543e-006*T_1_*T_2_^4^+7.051e-007*T_2_^5^
	Quintic+BP-ANN	0.9507	Y = −0.1454+6.803*T_1_−3.249*T_2_−67.57*T_1_^2^+66.13*T^1^*T_2_−26.76*T_2_^2^+192.9*T_1_^3^−132.7*T_1_^2^*T_2_−75.01*T_1_*T_2_^2^+99.66*T_2_^3^−198.7*T_1_^4^−14.87*T_1_^3^*T_2_+537.8*T_1_^2^*T_2_^2^−577.8*T_1_*T_2_^3^+155*T_2_^4^+67.97*T_1_^5^+78.26*T_1_^4^*T_2_−403.7*T_1_^3^*T_2_^2^+414.8*T_1_^2^*T_2_^3^−<1?show=[sr]?>104.4*T_1_*T_2_^4^−13.94*T_2_^5^
Princess VII	Quadratic	0.6940	Y = −0.5152+0.0604*T_1_−0.01284*T_2_−0.0008349*T_1_^2^+<1?show=[sr]?>0.001594*T_1_*T_2_−0.001628*T_2_^2^
	Quintic+Bisquare	0.9191	Y = −0.4047+0.5009*T_1_−0.3331*T_2_−0.08726*T_1_^2^+0.06563*T^1^*T_2_−0.002549*T_2_^2^+0.004956*T_1_^3^−0.001118*T_1_^2^*T_2_−0.00565*T_1_*T_2_^2^+0.003324*T_2_^3^−0.0001053*T_1_^4^−0.0001238*T_1_^3^*T_2_+0.0005272*T_1_^2^*T_2_^2^−0.000455*T_1_*T_2_^3^+0.0001159*T_2_^4^+7.324e-007*T_1_^5^+2.591e-006*T_1_^4^*T_2_−8.149e-006*T_1_^3^*T_2_^2^+6.436e-006*T_1_^2^*T_2_^3^−4.407e-007*T_1_*T_2_^4^−7.708e-007*T_2_^5^
	Quintic+BP-ANN	0.9439	Y = −0.6384+24.43*T_1_−13.54*T_2_−155.9*T_1_^2^+87.77*T^1^*T_2_+6.161*T_2_^2^+356.5*T_1_^3^−117*T_1_^2^*T_2_−165.9*T_1_*T_2_^2^+75.58*T_2_^3^−<1?show=[sr]?>322.3*T_1_^4^−109.2*T_1_^3^*T_2_+725.6*T_1_^2^*T_2_^2^−663.2*T_1_*T_2_^3^+<1?show=[sr]?>212.7*T_2_^4^+99.58*T_1_^5^+134.6*T_1_^4^*T_2_−482.2*T_1_^3^*T_2_^2^+432.9*T_1_^2^*T_2_^3^−94.28*T_1_*T_2_^4^−31.4*T_2_^5^

Contour plot maps were then used to visualize the prediction errors generated from different simulation functions (Fig. 2). The blue spot represents the experimental germination percentage associated with particular fluctuating temperature combinations. Obviously, DQEM is the one most prone to produce prediction errors among the compared models (Fig. 2A, D and G). However, some weakness in functional convergence of quintic equations was observed, a small portion of observed temperature-germination spots were still out of their equation derived surfaces (Fig. 2B, C, E, F, H and I).

### Spatial Mapping of Optimal Planting Times

**Figure 3:**
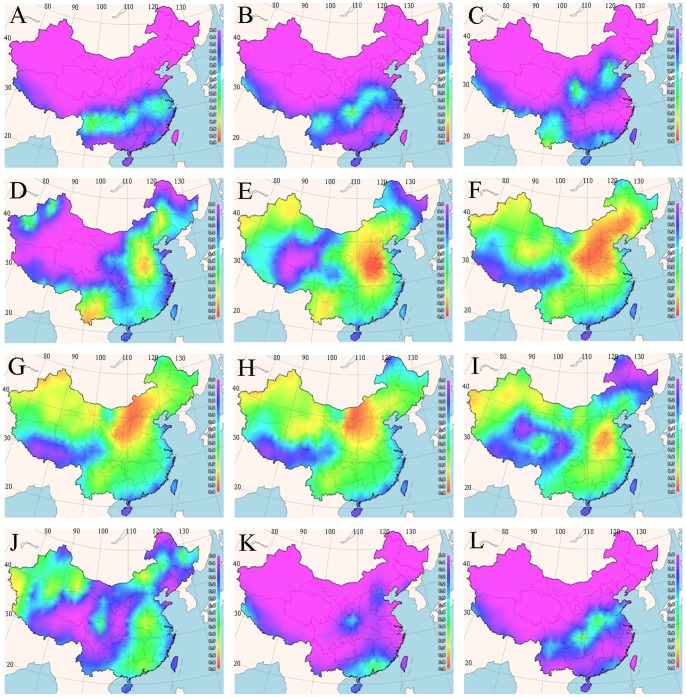
Maps of monthly germination suitability for *C. dactylon* in different regions of China; (A–L) January–December.

**Figure 4:**
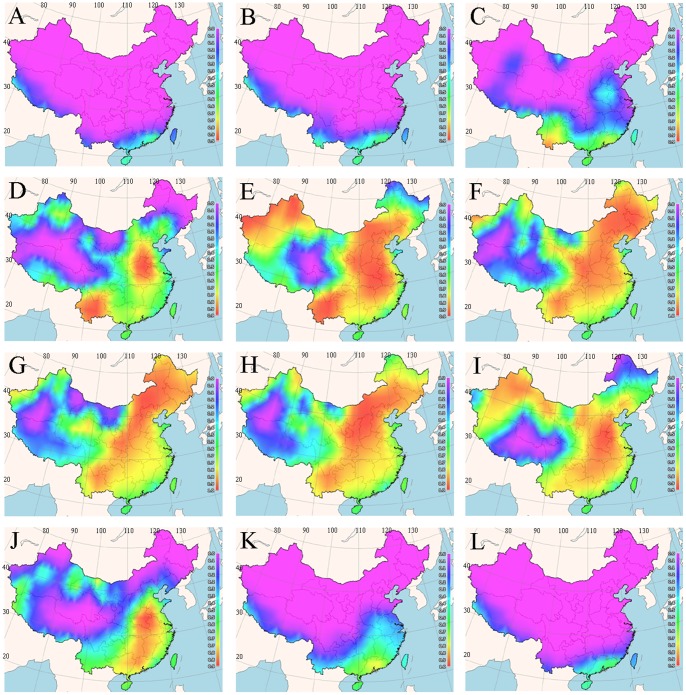
Maps of monthly germination suitability for ‘Savannah’ in different regions of China; (A–L) January–December.

**Figure 5:**
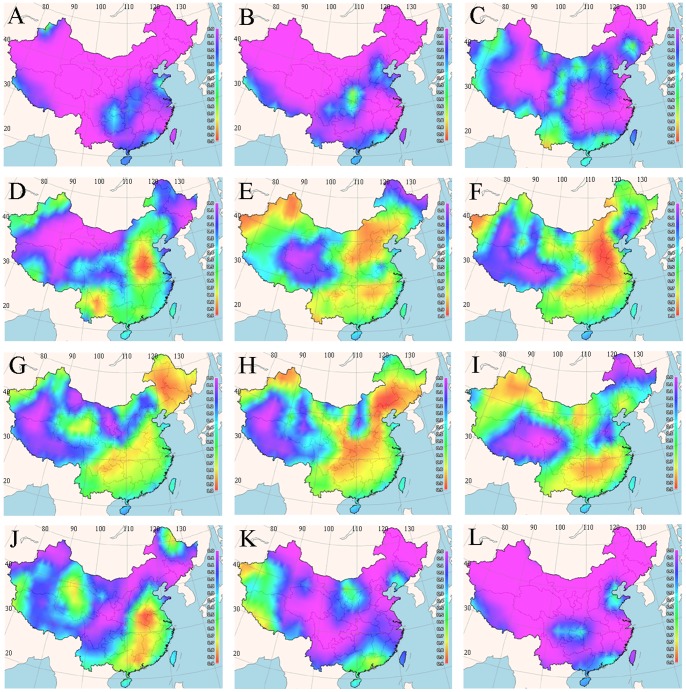
Maps of monthly germination suitability for ‘Princess VII’ in different regions of China; (A–L) January–December.

The temperature–germination functions derived from our experimental data were used to predict germination percentage for various regions in China in different seasons using the 25 years mean minimum and mean maximum earth surface temperature grids as input. The percentage reflected the likelihood of germination suitability at each grid cell of the maps *via* FreeMicaps (Fig. 3–5). The results reveal that most of the Chinese regions are not suitable for seed germination of all the tested bermudagrass cultivars from November to March. Although ‘Savannah’ has the narrowest geographic range of germination with percentages arranging from above 0 to 100%, it was predicted to have optimal temperatures for germination in widest geographic range in China (Fig. 4). In contrast, *C. dactylon* had the narrowest range for the optimal temperatures for seed germination (Fig. 3). For both *C. dactylon* and ‘Princess VII’, the widest range with the optimal temperatures for seed germination was in the month of June, whilst for ‘Savannah’ it was in May (Fig. 3–5).

## Discussion

Simulation models have been widely used to correlate cultivation conditions and plant germination/growth [38]–[40]. These regression models were generally used to predict the plant suitability to particular regions where climate and soil environment information is available. These mathematical models could help grass cultivation in three major aspects. First, these functions combined with a visual suitability map may help decision makers in selecting a grass species and planning its seeding timing [36], [41]. Second, they may serve as useful tools for identifying and evaluating desirable quantitative characteristics for specific grass breeding objectives [42], which is helpful in coupling genotype and phenotype of a target cultivar, a technology with significant application value in the rapidly expanding turf industry [43]. Third, these grass-condition models may improve our understanding of how changes in agricultural systems would quantitatively influence grass germination/growth [44].

### Comparison of Regressions Using Quadratic and Quintic Equations

The quadratic equation was broadly accepted as an effective statistical tool for simulating continuous variable-response relationship, and quadratic response surface has been a predominant method to analyze germination performance of grass seeds under various temperature regimes, especially to test the seed germination affected by diurnal temperature treatments [36], [41]. However, the weakness of quadratic equation is also obvious. Since the two-dimensional quadratic response surface cannot display some errors in the global fitting between quadratic function and experimental data [36], the weakness of low R^2^ values (0.61–0.80) was ignored. In this study, quintic equation was employed for regression to simulate diurnal temperature-germination responses of three bermudagrass cultivars. Compared to quadratic surfaces, quintic approaches had significantly lower fitting errors and higher confidence (Fig. 2 and Table 5). This may be due to the nonlinear temperature-germination correlation. Hence these quintic equation models could provide more reliable predictions for field performance of grasses, further improving the reliability of the suitability map of grasses tinted with different color based on predicted germination percentage data.

### BP-ANN and Bisquare for Intercept and Coefficients Fitting

Artificial Neural Network (ANN), an algorithm for simulating the thinking processes of the human brain which usually have multiple networks that are logically arranged as fundamental units [45]. The information of any unit can be learnt, recalled, concluded and speculated. Hence, the ANN has many advantages such as distributed storage of information, self-adaptability, self-organization and fault-tolerance properties [40]. As a computer based program, it could be used to perform large-scale parallel calculations to simulate nonlinear correlation [46]. Therefore, ANN is broadly used in various of fields including biomedical research [47], [48], optimization of soil nutrient distribution coefficient [43], [49], [50], forecasting of microorganism community assemblages [51], prediction of animal metabolism and diets correlation [52], and simulation of fruit post-ripening process [53]–[55].

Currently, there are tens of ANN models. Amongst them, the back propagation (BP) network is the most widely used one for simulation of nonlinear relationship [56]. The BP-ANN model belongs to supervised study and its training process has two phases, forward propagation and backward propagation [57]. In the forward propagation, the weighted value and threshold value of each layer is calculated by iteration and passed into the BP three-layer network. Subsequently, the backward propagation uses the weighted value and threshold values for revision [58]. These two phases commonly occur repeatedly for about 10, 000 times, and the weighted value and threshold values alternate until they converge. The target of these training processes is to generate a function that can globally ‘distinguish’ and ‘remember’ all the input raw data [59]. Therefore, the simulated BP-ANN functions could be used to predict proposed parameter with appropriate input variables.

The robust bisquare estimator, also known as Tukey's bi-weight function, was a popular choice for nonlinear function fitting [60]. This estimator was often used for smoothing the nonlinear response surface [61], [62]. It was reported that bisquare estimator could be adapted to process noisy data with outliers [63].

In our study, both BP-ANN and bisquare methods were used to optimize the temperature-germination functions for three bermudagrass cultivars. We did not find significant difference between general quintic functions and bisquare fitted quintic functions. Furthermore, most bisquare quintic functions in this study had slightly lower R^2^ values than the quintic functions. Therefore, the BP-ANN optimized quintic equations were found to be the best option for fitting temperature-germination functions of the tested bermudagrass cultivars.

To some degree, the BP-ANN based temperature-germination functions could be evaluated by the visualized suitability map (Figs. 3–5) and the observed temperature-germination results (Tables 1–3). Generally, the cultivar “Savannah” could germinate under the widest temperature regimes (Tables 1–3), from about 20/5°C to 40/40°C, it also present the highest suitability with widest geographic regions in China (eg. Fig. 4D–I). On the other hand, the predicted suitability map worked in concert with the observations that all the three cultivars preferred warm temperature above 15/15°C (Tables 1–3), and little germination could be found during the cool seasons in China (eg. Fig. 4A–C).

## Conclusions

This study tested the influence of diurnal fluctuations of temperatures on seed germination of three bermudagrass cultivars (*C. dactylon*, ‘Savannah’ and ‘Princess VII’). Eight temperature regimes, 5/30, 10/30, 5/35, 10/35, 15/35, 20/35, 15/40 and 20/40°C, supported the optimal temperature for seed germination for the three cultivars. However, the germination percentage of all the three cultivars was lower than 50% under conditions of constant temperatures ranging from 5 to 40°C.

To simulate the grass germination-temperature response function, both quadratic and quintic equations were employed. Quintic functions performed significantly better (R^2^ were around 0.9439) than the quadratic ones (R^2^ ranging from 0.6940∼0.8177) for the tested cultivars. The main objective of this study was to test the nonlinear fittin\g approaches, bisquare and BP-ANN, for optimizing the regression functions. Our results suggested that BP-ANN has significant advantages over bisquare for fitting the intercept and coefficients of the temperature-germination functions.

Based on the experimentally derived BP-ANN functions and available climate data, we prepared a seed suitability map of three bermudagrass cultivars for cultivation in the People’s Republic of China. We observed that most of the regions in China are not suitable for bermudagrass seed germination from November to March. The cultivar ‘Savannah’ had the widest geographic range of the optimal temperature for seed germination, whilst *C. dactylon* had the narrowest range of the optimal temperature for seed germination percentage. The month with widest range of the optimal temperature for seed germination for *C. dactylon* and ‘Princess VII’ is June, whilst for ‘Savannah’, it is May.

## Materials and Methods

### Seeds and Conditioning

The bermudagrass cultivars (*C. dactylon*, ‘Savannah’ and ‘Princess VII’) were planted widely in China. All the grass seeds were purchased from Shanghai Chunyin Turf Inc., (Shanghai, China) and stored at room temperature until use. Seed viability was tested on sterilized wet filter paper at 25°C in darkness [36].

Briefly, seeds were surface sterilized in 0.01% HgCl_2_ for 1 min followed by four rinses with distilled water. The seeds were subsequently placed on wet filter papers in Petri dishes. The Petri dishes were placed in incubators set up for 36 different regimes of diurnal fluctuations of temperature treatments: 16 h at temperature T_1_ and 8 h at temperature T_2_. The T_1_ and T_2_ ranged from 5 to 40°C with 5°C increments [36]. Germinated seeds were counted daily until no further germination occurred (about 15–20 days) for viability analysis (Supplementary file Tables 1–3). For each experiment, three replications of 50 seeds were used in a randomized block design.

### Statistical Analysis

In this study, quadratic and quintic response surfaces were constructed with estimated means and confidence intervals [64]. The quadratic equations were first used for estimating germination percentages. The generalized equation was [36]:


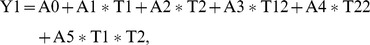


where Y_1_: predicted germination percentage, A_0_: intercept, A_1_ through A_5_: coefficients, T_1_ and T_2_: two temperature in the diurnal regime.

Meanwhile, quintic equations with the following generalized equation were also used to simulate the temperature-germination function: 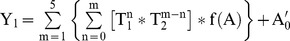, where A_0_^′^: intercept, f(A): coefficient function. Temperature inputs were normalized by dividing the maximum value. Subsequently, the intercept and coefficients were optimized using Bisquare, a default curve fitting method of MATLAB 7.9-R2009b software and back propagation artificial neural networks (BP-ANN) approach described in Fig. 1, respectively. The BP-ANN (or feed-forward network) has the capability to learn arbitrary nonlinearity and great potential for adaptive control applications [46]. In the BP-ANN, the correlations among the input variables do not need to be specified. Instead, they learn from the examples fed to them (Fig. 1). In addition, they can generalize correct responses that only broadly resemble the data in the learning phase [56], [65], [66].

### Spatial Mapping

The suitability of a grass is represented by seed germination percentage. The grass suitability maps were created using the FreeMicaps software (http://bbs.121323.com/guojf/FreeMicaps20111001.rar). Like the Surfer software [67], FreeMicaps also uses grid data at selected points (station) that is compatible with GIS. The temperature data of adjacent regions around the station are generated using regression functions. The data used to construct the temperature grid are curated in the database in National Aeronautics and Space Administration (NASA, http://power.larc.nasa.gov/cgi-bin/cgiwrap/solar/sse.cgi?grid@larc.nasa.gov#s11) which are composed of minimum and maximum daily temperature of earth surface from 313 Chinese weather stations for a period of 25 years (from 1983 to 2007). These mean values of minimum and maximum daily temperatures were used as the T_1_ and T_2_ variables, respectively in the BP-ANN-Quintic functions for calculating germination percentages (grass suitability).

## Supporting Information

Table S1 **Cumulative seed germination of amount** ***C. dactylon*** **at different days in 36 temperature regimes (50 seeds in total).**
(XLS)

Table S2 **Cumulative seed germination amount of Savannah at different days in 36 temperature regimes (50 seeds in total).**
(XLS)

Table S3 **Cumulative seed germination amount of Princess VII at different days in 36 temperature regimes (50 seeds in total).**
(XLS)

## References

[1] Farsani TM, Etemadi N, Sayed-Tabatabaei BE, Talebi M (2012) Assessment of Genetic Diversity of Bermudagrass (*Cynodon dactylon*) Using ISSR Markers. Int J Mol Sci 13: 383–392. 22312259 10.3390/ijms13010383 PMC3269693

[2] Huang CQ, Huang DY, Zhang YF, Liu GD (2010) Genetic analysis for 57 accessions of Cynodon dactylon from 17 countries in 5 continents by SRAP markers. Trop Grasslands 44: 274–281.

[3] Caglar BK, Satar S, Elbeaino T (2013) Detection and Molecular Characterization of Bermuda Grass (*Cynodon dactylon*) White Leaf Phytoplasma from Turkey. Int J Agric Biol 15: 90–94.

[4] Hameed M, Ashraf M, Naz N, Nawaz T, Batool R, et al (2013) Anatomical Adaptations of *Cynodon Dactylon* (L.) Pers. From the Salt Range (Pakistan) to Salinity Stress. Ii. Leaf Anatomy. Pak J Bot 45: 133–142.

[5] Heshmati GA, Pessarakli M (2011) Threshold Model in Studies of Ecological Recovery in Bermudagrass (*Cynodon Dactylon* L.) under Nutrient Stress Conditions. J Plant Nutr 34: 2183–2192.

[6] Adebiyi FM, Ayeni OA (2010) Evaluation of Phytoaccumulation of Selected Metals from Petroleum Products Impacted-Soils by *Cynodon dactylon* Plants Using AAS/AES Analytical Techniques. Anal Lett 43: 1879–1888.

[7] Castillo AM, Tercero TM, Davis JM (2009) Biolocalization of lead and cadmium in *Bouteloua curtipendula* and *Cynodon dactylon*. Abstr Pap Am Chem S 237.

[8] Gayathri U, Venkatraman BR, Arivoli S (2011) Removal of Copper (II) Ions from Aqueous Solutions by Adsorption with Low Cost Acid Activated *Cynodon Dactylon* Carbon. E-J Chem 8: S377–S391.

[9] Wang YB, Zhang L, Yao J, Huang YJ, Yan M (2009) Accumulation and Resistance to Copper of Two Biotypes of Cynodon dactylon. B Environ Contam Tox 82: 454–459. 10.1007/s00128-009-9640-9 19165407

[10] Wu FY, Bi YL, Leung HM, Ye ZH, Lin XG, et al (2010) Accumulation of As, Pb, Zn, Cd and Cu and arbuscular mycorrhizal status in populations of *Cynodon dactylon* grown on metal-contaminated soils. Appl Soil Ecol 44: 213–218.

[11] Dhoke V, Mishra A, Vohra R, Ghosh R, Kadam VJ (2008) Pharmacological evaluation for anti-ulcer effect of Cynodon dactylon pers. Against gastric ulcers in rats. Indian J Pharmacol 40: 69–70.21279169

[12] Garg VK, Paliwal SK (2011) Anti-Inflammatory Activity of Aqueous Extract of Cynodon dactylon. Int J Pharmacol 7: 370–375.

[13] Arjunan N, Murugan K, Madhiyazhagan P, Kovendan K, Prasannakumar K, et al (2012) Mosquitocidal and water purification properties of *Cynodon dactylon*, *Aloe vera*, *Hemidesmus indicus* and *Coleus amboinicus* leaf extracts against the mosquito vectors. Parasitol Res 110: 1435–1443. 21947308 10.1007/s00436-011-2646-3

[14] Balasubramanian G, Sarathi M, Venkatesan C, Thomas J, Hameed ASS (2008) Oral administration of antiviral plant extract of *Cynodon dactylon* on a large scale production against White spot syndrome virus (WSSV) in Penaeus monodon. Aquaculture 279: 2–5.

[15] Kaleeswaran B, Ilavenil S, Ravikumar S (2011) Dietary supplementation with *Cynodon dactylon* (L.) enhances innate immunity and disease resistance of Indian major carp, Cat la catla (Ham.). Fish Shellfish Immun 31: 953–962. 10.1016/j.fsi.2011.08.013 21888977

[16] Mangathayaru K, Umadevi M, Reddy CU (2009) Evaluation of the immunomodulatory and DNA protective activities of the shoots of *Cynodon dactylon* . J Ethnopharmacol 123: 181–184. 19429359 10.1016/j.jep.2009.02.036

[17] Babu DSR, Neeharika V, Pallavi V, Reddy BM (2009) Antidiarrheal activity of *Cynodon Dactylon*. pers. Pharmacogn Mag 5: 23–27.

[18] Rad AK, Hadjzadeh MAR, Rajaei Z, Mohammadian N, Valiollahi S, et al (2011) The Beneficial Effect of *Cynodon Dactylon* Fractions on Ethylene Glycol-Induced Kidney Calculi in Rats. Urol J 8: 179–184. 21910095

[19] Pal D (2008) Evaluation of CNS activities of aerial parts of *Cynodon dactylon* Pers. in mice. Acta Pol Pharm 65: 37–43. 18536171

[20] Pal DK, Kumar M, Chakraborty P, Kumar S (2008) Evaluation of the antioxidant activity of aerial parts of *Cynodon dactylon* . Asian J Chem 20: 2479–2481.

[21] Devi KMS, Annapoorani S, Ashokkumar K (2011) Hepatic antioxidative potential of ethyl acetate fraction of *Cynodon dactylon* in Balb/c mice. J Med Plants Res 5: 992–996.

[22] Rai DK, Sharma RK, Rai PK, Watal G, Sharma B (2011) Role of aqueous extract of *Cynodon dactylon* in prevention of carbofuran-induced oxidatives stress and acetylcholinesterase inhibition rat brain. Cell Mol Biol 57: 135–142. 21366973

[23] Sindhu G, Ratheesh M, Shyni GL, Helen A (2009) Inhibitory effects of *Cynodon dactylon* L. on inflammation and oxidative stress in adjuvant treated rats. Immunopharm Immunot 31: 647–653. 10.3109/08923970902947325 19874236

[24] Garcia-Mozo H, Galan C, Belmonte J, Bermejo D, Candau P, et al (2009) Predicting the start and peak dates of the Poaceae pollen season in Spain using process-based models. Agr Forest Meteorol 149: 256–262.

[25] Recio M, Docampo S, Garcia-Sanchez J, Trigo MM, Melgar M, et al (2010) Influence of temperature, rainfall and wind trends on grass pollination in Malaga (western Mediterranean coast). Agr Forest Meteorol 150: 931–940.

[26] van Oort PAJ, Zhang TY, de Vries ME, Heinemann AB, Meinke H (2011) Correlation between temperature and phenology prediction error in rice (*Oryza sativa* L.). Agr Forest Meteorol 151: 1545–1555.

[27] Bradford KJ (2002) Applications of hydrothermal time to quantifying and modeling seed germination and dormancy. Weed Sci 50: 248–260.

[28] Hardegree S, Van Vactor S (1999) Predicting germination response of four cool-season range grasses to field-variable temperature regimes. Environ Exp Bot 41: 209–217.

[29] Hardegree SP, Van Vactor SS, Pierson FB, Palmquist DE (1999) Predicting variable-temperature response of non-dormant seeds from constant-temperature germination data. Journal of Range Management: 83–91.

[30] Hardegree SP (2006) Predicting germination response to temperature. I. Cardinal-temperature models and subpopulation-specific regression. Annals of Botany 97: 1115–1125. 16624846 10.1093/aob/mcl071 PMC2803400

[31] Hardegree SP (2006) Predicting germination response to temperature. III. Model validation under field-variable temperature conditions. Ann Bot-London 98: 827–834. 10.1093/aob/mcl163 PMC2806169 16870642

[32] Hardegree SP, Winstral AH (2006) Predicting germination response to temperature. II. Three-dimensional regression, statistical gridding and iterative-probit optimization using measured and interpolated-subpopulation data. Ann Bot-London 98: 403–410. 10.1093/aob/mcl112 PMC2803456 16735412

[33] Roberts E (1988) Temperature and seed germination. Symposia of the Society for Experimental Biology. 109. 3077854

[34] Thompson K, Grime J, Mason G (1977) Seed germination in response to diurnal fluctuations of temperature. Nature 267: 147–149. 16073423 10.1038/267147a0

[35] Vandelook F, Van Assche JA (2008) Temperature requirements for seed germination and seedling development determine timing of seedling emergence of three monocotyledonous temperate forest spring geophytes. Ann Bot-London 102: 865–875. 10.1093/aob/mcn165 PMC2712388 18757880

[36] Lu H, Shen J, Jin X, Hannaway DB, Daly C, et al (2008) Determining optimal seeding times for tall fescue using germination studies and spatial climate analysis. Agr Forest Meteorol 148: 931–941.

[37] Daly C, Gibson WP, Taylor GH, Johnson GL, Pasteris P (2002) A knowledge-based approach to the statistical mapping of climate. Clim Res 22: 99–113.

[38] Gazola S, Scapim CA, Guedes TA, Braccini AdL (2011) Nonlinear proposal modeling of seed germination performance of hybrid corn seeds. Ciência Rural 41: 551–556.

[39] Onofri A, Carbonell E, PIEPHO HP, Mortimer A, Cousens R (2010) Current statistical issues in Weed Research. Weed Res 50: 5–24.

[40] Shetty N, Olesen MH, Gislum R, Deleuran LC, Boelt B (2012) Use of partial least squares discriminant analysis on visible-near infrared multispectral image data to examine germination ability and germ length in spinach seeds. J Chemometr 26: 462–466.

[41] Shen J, Xu L, Jin X, Chen J, Lu H (2008) Effect of temperature regime on germination of seed of perennial ryegrass (Lolium perenne). Grass Forage Sci 63: 249–256.

[42] Rasul G, Humphreys GD, Wu J, Brûlé-Babel A, Fofana B, et al (2012) Evaluation of preharvest sprouting traits in a collection of spring wheat germplasm using genotype and genotype × environment interaction model. Plant Breeding 131: 244–251.

[43] Falamaki A (2013) Artificial neural network application for predicting soil distribution coefficient of nickel. J Environ Radioactiv 115: 6–12. 10.1016/j.jenvrad.2012.06.008 22846874

[44] Rivera-Reyes J, Peraza-Luna F, Serratos-Arévalo J, Posos-Ponce P, Guzmán-Maldonado S, et al (2009) Effect of nitrogen and phosphorus fertilization on phytic acid concentration and vigor of oat seed (var. Saia) in Mexico. Phyton (Buenos Aires) 78: 37–42.

[45] Evrendilek F (2013) Quantifying biosphere―atmosphere exchange of CO2 using eddy covariance, wavelet denoising, neural networks, and multiple regression models. Agr Forest Meteorol 171: 1–8.

[46] Catalogna M, Cohen E, Fishman S, Halpern Z, Nevo U, et al (2012) Artificial neural networks based controller for glucose monitoring during clamp test. PloS one 7: e44587. 22952998 10.1371/journal.pone.0044587 PMC3432111

[47] Akdemir B, Oran B, Gunes S, Karaaslan S (2009) Prediction of aortic diameter values in healthy turkish infants, children, and adolescents by using artificial neural network. J Med Syst 33: 379–388. 19827264 10.1007/s10916-008-9200-6

[48] Alam M, Briggs A (2011) DA1 Artificial Neural Network Meta-Models in Cost-Effectiveness analysis of intensive Blood-Glucose Control: A Case Study Applied to the UK prospective Diabetes Study (UKPDS) Individual Patient outcome Simulation Model. Value Health 14: A234–A235.

[49] Adamowski J, Chan HF, Prasher SO, Ozga-Zielinski B, Sliusarieva A (2012) Comparison of multiple linear and nonlinear regression, autoregressive integrated moving average, artificial neural network, and wavelet artificial neural network methods for urban water demand forecasting in Montreal, Canada. Water Resour Res 48.

[50] Alvarez R, Steinbach H, Bono A (2011) An artificial neural network approach for predicting soil carbon budget in agroecosystems. Soil Sci Soc Am J 75: 965–975.

[51] Larsen PE, Field D, Gilbert JA (2012) Predicting bacterial community assemblages using an artificial neural network approach. Nat Methods 9: 621–625. 22504588 10.1038/nmeth.1975

[52] Ahmadi H, Golian A (2010) Growth analysis of chickens fed diets varying in the percentage of metabolizable energy provided by protein, fat, and carbohydrate through artificial neural network. Poultry Sci 89: 173–179. 20008816 10.3382/ps.2009-00125

[53] Poonnoy P, Tansakul A, Chinnan M (2007) Artificial Neural Network Modeling for Temperature and Moisture Content Prediction in Tomato Slices Undergoing Microwave-Vacuum Drying. J Food Sci 72: E042–E047. 17995884 10.1111/j.1750-3841.2006.00220.x

[54] Torrecilla JS, Mena ML, Yáñez-Sedeño P, García J (2007) Quantification of phenolic compounds in olive oil mill wastewater by artificial neural network/laccase biosensor. J Agr Food Chem 55: 7418–7426. 17685539 10.1021/jf0703351

[55] Zheng H, Jiang L, Lou H, Hu Y, Kong X, et al (2010) Application of artificial neural network (ANN) and partial least-squares regression (PLSR) to predict the changes of anthocyanins, ascorbic acid, Total phenols, flavonoids, and antioxidant activity during storage of red bayberry juice based on fractal analysis and red, green, and blue (RGB) intensity values. J Agr Food Chem 59: 592–600. 21190362 10.1021/jf1032476

[56] Zhaoa X, Xua K, Shia H, Chengb J, Mac J, et al (2013) Application of the back-error propagation artificial neural network (BPANN) on genetic variants in the PPAR-γ and RXR-α gene and risk of metabolic syndrome in a Chinese Han population. J Biomed Res 27 doi:10.7555/JBR.27.20120061 10.7555/JBR.27.20120061 PMC3968282 24683409

[57] Karnin ED (1990) A simple procedure for pruning back-propagation trained neural networks. Neural Networks, IEEE Transactions on 1: 239–242. 10.1109/72.80236 18282841

[58] Chang W, Bosworth B, Carter GC (1993) Empirical results of using back-propagation neural networks to separate single echoes from multiple echoes. Neural Networks, IEEE Transactions on 4: 993–995. 10.1109/72.286895 18276530

[59] Pan Y, Jiang J, Wang Z (2007) Quantitative structure–property relationship studies for predicting flash points of alkanes using group bond contribution method with back-propagation neural network. J Hazard Mater 147: 424–430. 17292543 10.1016/j.jhazmat.2007.01.025

[60] Omelka M, Salibián-Barrera M (2010) Uniform asymptotics for S-and MM-regression estimators. Ann I Stat Math 62: 897–927.

[61] Hampel F, Hennig C, Ronchetti E (2011) A smoothing principle for the Huber and other location M-estimators. Comput Stat ata An 55: 324–337.

[62] Ji J (2012) Robust inversion using biweight norm and its application to seismic inversion. Explor Geophys 43: 70–76.

[63] Maddahi A, Shahriari H, Shokouhi AH (2011) A robust (X)over-bar control chart based on M-estimators in presence of outliers. Int J Adv Manuf Tech 56: 711–719.

[64] Barnhart B, Eichinger W, Prueger J (2012) Introducing an Ogive method for discontinuous data. Agr Forest Meteorol 162: 58–62.

[65] Hema M, Srinivasan K (2011) Artificial neural network and multiple regression model for nickel (II) adsorption on powdered activated carbons. Journal of Environmental Science & Engineering 53: 237. 23029923

[66] Yun J, Mackenzie M, Rathee S, Robinson D, Fallone B (2012) An artificial neural network (ANN)-based lung-tumor motion predictor for intrafractional MR tumor tracking. Med Phys 39: 4423. 22830775 10.1118/1.4730294

[67] Modi Y, De Beer D, Agrawal S (2011) Physical modelling of terrain directly from surfer grid and ARC/INFO ASCII data formats. S Afr J Ind Eng 23: 230–241.

